# Gemcitabine combined with continuous infusion 5-fluorouracil in advanced and symptomatic pancreatic cancer: a clinical benefit-oriented phase II study

**DOI:** 10.1054/bjoc.1999.1139

**Published:** 2000-06

**Authors:** E Matano, P Tagliaferri, A Libroia, V Damiano, A Fabbrocini, S De Lorenzo, A R Bianco

**Affiliations:** Cattedra di Oncologia Medica, Dipartimento di Endocrinologia ed Oncologia Molecolare a Clinica, Facoltà di Medicina e Chirurgia, Università “Federico II”, Via S. Pansini 5, Napoli, 80131, Italy

**Keywords:** advanced pancreatic cancer, phase II study, gemcitabine, 5-fluorouracil, clinical benefit response

## Abstract

Gemcitabine and 5-fluorouracil are the only two compounds with reproducible activity against advanced pancreatic cancer (APC). We have evaluated a novel combination of gemcitabine and 5-fluorouracil on the clinical benefit response (CBR) end point. Eleven consecutive patients with symptomatic APC were entered in a two-stage phase II trial. Gemcitabine was administered by intravenous (i.v.) bolus injection at the dose of 1000 mg m^−2^on days 1, 8, 15 and 5-fluorouracil 500 mg m^−2^was given by continuous i.v. infusion on days 1–5. Treatment was repeated every 28 days. A CBR was achieved in 7/11 patients. The mean time to loss of CBR was 26.5 weeks (range 14–18, median 22). Toxicity was mild and no APC patient experienced WHO grade 3 toxicity. The gemcitabine/5-fluorouracil combination is well tolerated and produces a symptomatic relief in the majority of APC patients. © 2000 Cancer Research Campaign


*British Journal of Cancer* (2000) **82**(11), 1772–1775
© 2000 Cancer Research Campaign
DOI: 10.1054/ bjoc.1999.1139, available online at http://www.idealibrary.com on IDEAL®

### Gemcitabine combined with continuous infusion 5-fluorouracil in advanced and symptomatic pancreatic cancer: a clinical benefit-oriented phase II study

**E Matano, P Tagliaferri*, A Libroia, V Damiano, A Fabbrocini, S De Lorenzo and AR Bianco**

Cattedra di Oncologia Medica, Dipartimento di Endocrinologia ed Oncologia Molecolare a Clinica, Facoltà di Medicina e Chirurgia, Università "Federico II", Via S. Pansini 5, 80131 Napoli, Italy

**Summary** Gemcitabine and 5-fluorouracil are the only two compounds with reproducible activity against advanced pancreatic cancer (APC). We have evaluated a novel combination of gemcitabine and 5-fluorouracil on the clinical benefit response (CBR) end point. Eleven consecutive patients with symptomatic APC were entered in a two-stage phase II trial. Gemcitabine was administered by intravenous (i.v.) bolus injection at the dose of 1000 mg $m^{-2}$ on days 1, 8, 15 and 5-fluorouracil 500 mg $m^{-2}$ was given by continuous i.v. infusion on days 1–5. Treatment was repeated every 28 days. A CBR was achieved in 7/11 patients. The mean time to loss of CBR was 26.5 weeks (range 14–18, median 22). Toxicity was mild and no APC patient experienced WHO grade 3 toxicity. The gemcitabine/5-fluorouracil combination is well tolerated and produces a symptomatic relief in the majority of APC patients. © 2000 Cancer Research Campaign

**Keywords:** advanced pancreatic cancer; phase II study; gemcitabine; 5-fluorouracil; clinical benefit response

Surgically unresectable advanced pancreatic cancer (APC) has an extremely poor prognosis and is often highly symptomatic. The unfavourable outcome of APC has not been substantially modified by systemic treatment. 5-Fluorouracil (5-FU)-based chemotherapy has been extensively used in APC. A variety of different schedules and regimens have been evaluated and consistent anti-tumour activity has been reported in a minority of patients. Symptomatic relief was not readily assessed in the majority of these studies (Crown et al, 1991; Rubin et al, 1996). Gemcitabine (2′2′-difluor-deoxycytidine) is a deoxycytidine analogue with high activity in preclinical models of solid tumours and in human cloning assays (Von Hoff, 1996). Gemcitabine has anti-tumour clinical activity in lung cancer and pancreatic cancer. In the latter tumour, gemcitabine produces an 11% response rate and symptomatic relief in a substantially larger percentage of patients (Burris et al, 1995; Noble and Goa, 1997). New criteria of treatment effectiveness have been recently defined in APC considering that (a) the standard activity criteria based on tumour regression are difficult to assess in the case of retroperitoneal disease and massive liver involvement, and (b) APC is often highly symptomatic with a rapid decline in performance status. Improvement of survival does not appear an easy task, based on the use of currently available drugs. The clinical benefit response (CBR) criteria have been designed for reproducible evaluation of gemcitabine activity in APC. CBR is defined as a structured algorithmic approach which is based on the composite measure of symptomatic improvement based on pain intensity, analgesic consumption, performance status and weight (Carmichael, 1997; Stephens, 1998). Burris et al have demonstrated that gemcitabine produced an increase in CBR and survival if compared to 5-FU in a randomized prospective trial (Burris et al, 1995). We have approached the treatment of APC combining gemcitabine with 5-FU given by continuous infusion. The rationale of this combination was based on the contemporary use of the two drugs that show anti-tumour activity in APC. Moreover, the use of gemcitabine and 5-FU was allowed by the different toxicity profile and by the potential advantages of combining two nucleoside inhibitors. The continuous infusion modality for 5-FU should reduce myelotoxicity, prolong thymidilate synthetase inhibition and increase the chance of pharmacodynamic interactions with gemcitabine. The study was based on the Simon's two-stage optimal design and accrual could be continued to the second stage only if a predetermined number of responses should be reached on the first stage based on a predefined target activity (Simon, 1989). CBR was selected as the primary end point for this study.

*Received 16 April 1999*
*Revised 9 November 1999*
*Accepted 22 December 1999*

*Correspondence to:* E Matano

### PATIENTS AND METHODS

### Patient accrual

Consecutive patients with histologically confirmed locally advanced or metastatic APC were eligible. Symptomatic disease was defined according to the following stringent criteria:

a. a performance status < 70 according to Karnovsky in the absence of concurrent illness
b. pain requiring analgesia on a daily basis; pharmacologic treatment should be quantified as morphine equivalent (equalized to mg/day morphine consumption)

*Present address: Dipartimento di Medicina Sperimentale e Clinica, Facoltà di Medicina e Chirurgia, Università "Magna Graecia", Via T. Campanella 115, 88100 Catanzaro, Italy c. Baseline pain intensity score of > or 20 mm (of a possible 100 mm on the Memorial Pain Assessment Card)
d. loss of > 10% of body weight.

Only patients with at least one assessable criterium were considered for accrual. Adequate organ function with creatinine < 2.0 mg dl$^{-1}$, bilirubin < 1.5 mg dl$^{-1}$, serum albumin > 2.5 mg dl$^{-1}$, transaminases < 2.5 × the upper limit of institutional standards, baseline absolute neutrophil count (ANC) > 1500 µl$^{-1}$ and platelet count > 100 000 µl$^{-1}$ were required. Patients with a PS < 50 or a life expectancy of < 3 months could not be included in the study. Additional exclusion criteria were symptomatic heart disease and central nervous involvement. After approval, all patients gave informed consent according to bioethical requirements.

### Treatment

Gemcitabine-hydrochloride (Gemzar, Eli Lilly) was administered by intravenous (i.v.) bolus injection at the dose of 1000 mg m$^{-2}$ on days 1, 8 and 15, and 5-FU (Roche) was given by continuous i.v. infusion on days 1–5 at the daily dose of 500 mg m$^{-2}$. Central venous catheters (CVC) were inserted in all patients. Treatment was repeated every 28 days. In the case of persistent neutropenia (ANC < 1000 µl$^{-1}$) or thrombocytopenia (platelets < 100 000 µl$^{-1}$), treatment was delayed until recovery to ANC > 1500 µl$^{-1}$ and platelets ≥ 150 000 µl$^{-1}$. A 50% gemcitabine and 50% 5-FU dose reduction was planned in the case of ANC 500–1000 µl$^{-1}$ and platelets 50 000–100 000 µl$^{-1}$ after 2 weeks delay. Colony stimulating factors (CSFs) were not included in the study design and could be considered only in the case of neutropenic fever or persistent grade 4 neutropenia. A pain stabilization lead in period of 7 days was allowed before beginning treatment for an accurate determination of basal values.

### Study design

A single institution phase II study was prospectively projected according to the Simon's two-stage optimal design (Simon, 1989). According to this design a number (n1) patients are entered in the first stage of the trial. The accrual continues to a total of n2 patients only if a specified r1 response rate is achieved in the first series. A target activity of 25% response rate with a lower activity of 5% have been selected, with a 0.05 $\alpha$ error and a 0.20 $\beta$ error. In this case the treatment under investigation should be considered non-active if it produced no responses out of nine consecutive patients in the first series and fewer than 4/30 patients in the overall series. Taking into account the specific features of APC, the primary end point of the study was the achievement of a CBR according to the previously described criteria and to the definition of toxicity profile (Burris et al, 1995; Carmichael, 1997; Noble et al, 1997; Stephens, 1998). Anti-tumour activity as defined by the standard criteria of tumour regression was the secondary end point of the study. Assessment of CBR was performed weekly according to the structured algorithm which has been developed in order to provide an alternate end point in clinical trials of symptomatic APC (Figure 1). An improvement of 50% from baseline provided a positive score for pain intensity, while a 50% reduction of basal analgesic consumption was classified as a positive response on this latter parameter. The algorithm considers change in pain, evaluated as changes in pain intensity and in analgesic consumption and changes in performance status as the primary measures of clinical benefit. A patient was classified positive on Karnovsky Performance Status (KPS) if showed an improvement of at least 20 points over the baseline maintained for at least 12 weeks. Change in weight is considered the secondary measure of clinical benefit and an increase of 7% over the baseline was considered as a positive response. CBR was designed in order to identify an improvement more than the stabilization of disease-related symptoms. Time to loss of CBR (TTL-CBR) was calculated as the time from beginning of chemotherapy to the loss of symptomatic improvement induced by the treatment. A pretreatment clinical evaluation was performed and was repeated every 3 weeks. Imaging procedures (CT scan, ultrasound and/or nuclear magnetic resonance) were routinely performed before starting treatment and every 6 weeks thereafter. Additional procedures were allowed at clinical judgement. Disease progression confirmed by imaging procedures allowed determination of progression-free survival (PFS). Tumour response was defined under the standard criteria.

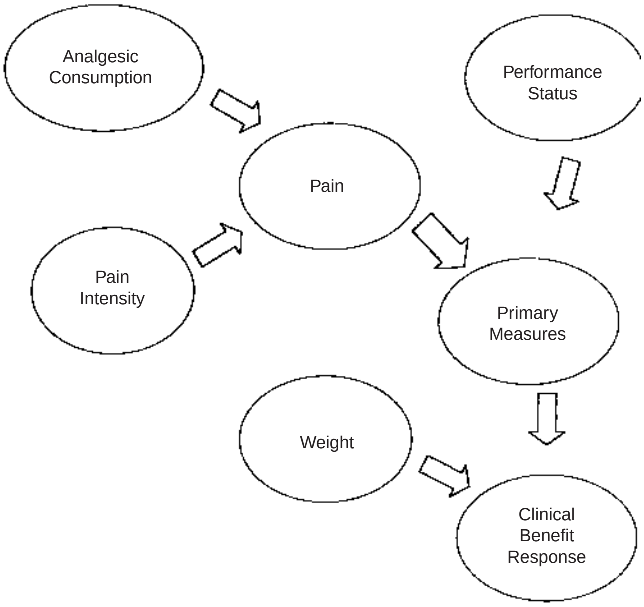

**Figure 1** Clinical benefit response algorithm

### RESULTS

Eleven consecutive patients with symptomatic APC were entered into the study and all were considered on intention-to-treat analysis; six patients were male and five female with a median age of 60.5 years (range 37–76). All patients had intra-abdominal disease. Liver metastasis were detected in 5/11 (45.4%) patients. A median of 5 monthly courses were given (range 3–8). Seven out of 11 (63.6%) patients were responders on pain assessments: four patients were classified positive both on pain intensity and analgesic consumption, two patients were positive on pain intensity and stable on analgesic consumption, one patient was positive on analgesic consumption and stable on pain intensity. In addition, three patients were stable on both parameters and only one patient presented worsening of pain and required an increase of analgesic dosage (Table 1A). Primary measures determination was subsequently performed: patients classified as responsive or stable on

**Table 1** Clinical Benefit Response assessment in the phase II study (number of patients)

| (A) | | Pain intensity | | | |
|---|---|---|---|---|---|
| | | Positive | Stable | Negative | Total |
| Analgesic | Positive | 4 | 1 | 0 | 5 |
| Consumption | Stable | 2 | 3 | 0 | 5 |
| | Negative | 0 | 0 | 1 | 1 |
| | Total | 6 | 4 | 1 | 11 |

| Pain | |
|---|---|
| Category | |
| Positive | 7 |
| Stable | 3 |
| Negative | 1 |

| (B) | | Performance status | | | |
|---|---|---|---|---|---|
| | | Positive | Stable | Negative | Total |
| Pain | Positive | 2 | 5 | 0 | 7 |
| | Stable | 0 | 3 | 0 | 3 |
| | Negative | 0 | 0 | 1 | 1 |
| | Total | 2 | 8 | 1 | 11 |

| Primary measures | |
|---|---|
| Category | |
| Positive | 7 |
| Stable | 3 |
| Negative | 1 |

| (C) | | Primary measures | | | |
|---|---|---|---|---|---|
| | | Positive | Stable | Negative | Total |
| Weight | Positive | 4 | 0 | 0 | 4 |
| | Non responsive | 3 | 3 | 1 | 7 |
| | Total | 7 | 3 | 1 | 11 |

| Clinical Benefit Response | |
|---|---|
| Category | |
| Responder | 7 |
| Non responder | 4 |

pain were assessed for KPS improvement. All patients classified positive on pain were also positive or stable for KPS. Seven out of 11 patients were therefore classified positive on primary measures and three were considered stable (Table 1B). Patients positive on primary measures were considered clinical benefit responders. Evaluation of body weight changes did not alter the CBR rate because none of the patients who were stable on primary measures showed a positive weight change and could be reclassified as clinical benefit responders (Table 1C). In conclusion, a CBR was experienced by 7/11 (63.6%) APC patients. This result exceeded the projected response rate and the gemcitabine/5-FU combination could be considered to provide a positive result on the primary endpoint of our trial. Under the standard tumour imaging criteria, 1/11 (9.09%) PR was achieved, while stable disease was recorded in 5/11 (45.4%), (Table 2). TTL-CBR could be a surrogate end point which should be considered a substitute for PFS in clinical benefit-oriented studies. In our study the mean duration of TTL-CBR was 26.5 weeks (range 14–48, median 22). The gemcitabine/5-FU combination was well tolerated and no APC patient experienced grade 3 toxicity. Grade 2 diarrhoea occurred in 2/11, grade 2 nausea and vomiting in 2/11 and grade 2 neutropenia in 5/11 patients. (Table 3) Neutropenic fever was never recorded and

**Table 2** Response to treatment

| | | | |
|---|---|---|---|
| CR (%) | 0/11 | CBR (%) | 7/11 (63.6) |
| PR (%) | 1/11 (9.09) | | |
| SD (%) | 5/11 (45.4) | | |
| DP (%) | 5/11 (45.4) | | |
| | PFS | TTL-CBR | |
| Mean (range) | 31.4 (16–44) | 26.5 (14–48) | |
| Median | 40 | 22 | |

CR, complete remission; PR, partial remission; SD, stable disease; DP, disease progression; CBR, clinical benefit response; PFS, progression-free survival; TTL-CBR, time to loss of clinical benefit response.

**Table 3** Number of patients who experienced toxic effects to gemcitabine/5-fluorouracil

| | Number of patients (%) | | | |
|---|---|---|---|---|
| **Toxicity** | **Grade 1** | **Grade 2** | **Grade 3** | **Grade 4** |
| Rash | 2 (18.18) | | | |
| Fever | 4 (36.36) | | | |
| Nausea/vomiting | 2 (36.36) | 2 (36.36) | | |
| Fatigue | 1 (9.09) | | | |
| Diarrhoea | 1 (9.09) | 2 (36.36) | | |
| Neutropenia | 1 (9.09) | 2 (36.36) | | |

CSFs were not required in this series of patients. The insertion of a CVC did not cause major complications.

### DISCUSSION

Our study demonstrates that the gemcitabine/5-FU combination under this schedule and dosages is well tolerated and induces symptomatic relief in the majority of APC patients. CBR was achieved in 7/11 (63.6%). The strict requirement for the CBR determination under a structured algorithm looks for a positive effect more than stabilization of the pre-existing status (Carmichael, 1997; Stephens, 1998). The CBR is, therefore, an effectiveness measure which appears suitable also as an end point for phase II trials in highly symptomatic APC. It is important to consider that only a major response under the standard criteria of tumour regression was observed in our study, and the gemcitabine/5-FU combination would have been rejected as inactive if the study had been designed with the standard response criteria as the primary end point. The CBR rate (7/11, 63.6%) which has been achieved in the first phase of a two-stage design, exceeded the minimal requirement (4/30) for demonstrating activity in the series, suggest that the final validation phase (second stage according to the Simon's design) could be performed as the experimental arm of a prospectively randomized study where gemcitabine monochemotherapy should be considered as the standard control arm. It has been reported that APC patients treated with gemcitabine alone achieved CBR in the 24% of cases and with 5-FU in the 5%. In this way the subsequent phase of gemcitabine/5-FU evaluation should benefit from the randomization process in order to avoid the phase II selection bias and over-estimation of results. Overall survival should become the primary end point with CBR and TTL-CBR as the secondary end points. Data on TTL-CBR have been compared with the conventional end point of PFS based on clinical evaluation and tumour imaging. In our series of

*© 2000 Cancer Research Campaign*

patients TTL-CBR, based on weekly assessment, provided earlier evidence of loss of therapeutical benefit as compared to the PFS; TTL-CBR mean was 26.5 weeks (range 14–48), while mean PFS was 31.4 weeks (range 16–44). Additional comparison between the two end points has now to be performed in larger series of APC patients. Quality of life assessment by standard approach such as the EORTC QLQ C-30 questionnaire should be also performed in parallel and provide a comparative analysis to CBR definition by the structured algorithm (Aaranson et al, 1993). The relationship between symptom relief and quality of life needs to be evaluated in a prospective trial. It is important to consider that parallel phase I–II studies of a similar combination where 5-FUu was given together with gemcitabine reported a 38.46–66% CBR demonstrating the highly symptomatic activity of this combination (Cascinu et al, 1997; Hidalgo et al, 1997). Notably the highest activity is reported when 5-FU was given by continuous i.v. infusion on days 1–5 at the daily dose of 500 mg $m^{-2}$. In conclusion our results indicate that gemcitabine/5-fluorouracil deserves further investigation and should be compared to standard gemcitabine monochemotherapy. Our findings underscore the need of alternate effectiveness end points in symptomatic tumours as APC were tumour regression and long-term survival are unlikely under the presently available therapeutic approaches.

### REFERENCES

Aaranson NK, Ahmedzai S, Bergman B et al (1993) The European Organization for Research and Treatment of Cancer QL C-30: a quality of life instrument for use in international clinical trials in Oncology. *J Natl Cancer Inst* **85**: 365–376

Burris H, Moore MJ, Andersen J et al (1995) Improvement in survival and clinical benefit with gemcitabine as first-line therapy for patients with Advanced Pancreatic Cancer: a randomized trial. *J Clin Oncol* **15**: 2403–2413

Carmichael J (1997) Clinical response benefit in patients with advanced pancreatic cancer: role of gemcitabine. *Digestion* **58**: 503–507

Cascinu S, Frontini L, Labianca R et al (1997) Gemcitabine (Gem) and 5-Fluorouracil (5-FU) in advanced pancreatic cancer, a GISCAD phase II study. *Proc ECCO* **33**: S280 (A1268)

Crown J, Casper ES, Biotet J et al (1991) Lack of efficacy of high dose leucovorin and 5 Fluorouracil in patients with advanced pancreatic adenocarcinoma. *J Clin Oncol* **8**: 1682–1686

Hidalgo M, Paz-Ares L and Hitt R (1997) Phase I–II study of gemcitabine combined with continuous infusion 5-fluorouracil as first-line chemotherapy in locally advanced and symptomatic pancreatic cancer. *Proc Am Soc Clin Oncol* **16**: A1030

Noble S and Goa KL (1997) Gemcitabine: a review of its pharmacology and clinical potential in non-small-cell lung cancer and pancreatic cancer. *Drugs* **54**: 447–472

Rubin J, Gallangher JG, Schroeder G et al (1996) Phase II trial of 5-fluorouracil and leucovorin in patients with metastatic gastric or pancreatic carcinoma. *Cancer* **78**: 1888–1891

Simon R (1989) Optimal two stage designs for phase II clinical trials. *Control Clin Trials* **10(1)**: 1–10

Stephens CD (1998) Gemcitabine: a new approach to treating pancreatic cancer. *Oncol Nurs Forum* **25**: 87–93

Von Hoff DD (1996) Activity of gemcitabine in a human cloning assay as a basis for clinical trials with gemcitabine. San Antonio Development Team. *Invest New Drug* **14**: 265–270

*© 2000 Cancer Research Campaign*
